# Big five personality and recreation specialization are related to satisfaction with life in birders

**DOI:** 10.1016/j.heliyon.2023.e21455

**Published:** 2023-10-23

**Authors:** Christoph Randler, Arash Rahafar, Nadine Großmann

**Affiliations:** a Department of Biology, Eberhard-Karls-University Tübingen, Tuebingen, Germany; b Department for Didactics of Biology, University of Cologne, Cologne, Germany

**Keywords:** Big five personality, Satisfaction with life, Birding, Leisure, Recreation specialization

## Abstract

Personality traits and recreation specialization are related to an individual's satisfaction with life. In addition, previous research has identified relationships between personality traits and recreational specialization. However, little is known about the interrelationship of the two variables in relation to life satisfaction. In this study, we addressed these relationships in birdwatchers. 555 birders (*M*_age_ = 49.11 years, *SD*_age_ = 17.14; 380 males, 170 females, 2 diverse, 3 without answer) from German-speaking countries filled out an online survey. The participants rated the BFI-10, a global assessment of life satisfaction, and three dimensions of recreation specialization (skill/knowledge, behavior, commitment). Satisfaction with life was positively correlated with extraversion, agreeableness, and conscientiousness, and negatively with neuroticism. Concerning recreation specialization, positive relationships between satisfaction with life and skill/knowledge as well as behavior were found; especially the dimension of skill/knowledge was positively related to conscientiousness and negatively to neuroticism. Openness was negatively related to the three dimensions of recreation specialization. The mediation analyses showed that skill/knowledge is directly and indirectly related to satisfaction with life. Birding as active outdoor activity has positive relationship with life satisfaction, and this seems to be mainly based on the cognitive component, with a high knowledge related to a higher life satisfaction.

## 1 Introduction

Personality traits on the one side and recreation specialization on the other, have both been found to be related with an individual's satisfaction with life [[1], [2], [3], [4]]. Concerning Big Five personality traits, individuals who score high in extraversion, agreeableness, openness, emotional stability, and conscientiousness report higher levels of life satisfaction compared to those who score low on these traits [2,5,6]. Moreover, perseverance of effort was positively associated with life satisfaction [1]. Concerning recreation specialization and leisure satisfaction, research has also shown that individuals who participate in leisure activities that match their interests and abilities experience greater life satisfaction [7,8]. Finally, the association between recreation specialization and personality has been addressed in a few studies [9]. These findings of previous studies suggest that both personality traits and recreation specialization can play an important role in shaping an individual's satisfaction with life. Therefore, we study the three variables life satisfaction, personality, and recreation specialization in a comprehensive manner to explore the relationships between them. In this study, we used the Big Five personality domains because it is one of the most widely used questionnaires of personality, and in addition, there are many published studies and even meta-analyses on the relationship between big five personality traits and life satisfaction. Birders provide a good example to study these relationships because birding comprises both, an outdoor activity in nature, which relates birding to sports, but also a cognitive component (e.g., identify bird species), which relates it to more cognitive recreational activities like chess.

### 1.1 Life satisfaction and big-five personality traits

Quality of life in societies is often assessed by economic and social indicators, but the advocates of subjective well-being measures argument that these provide only an incomplete picture, and that a subjective measurement of well-being is useful [10]. Subjective well-being consists of a cognitive and affective assessment of one's own life [11]. To study individual differences, a subjective individual measure is needed because nationwide indicators may not be sufficient to explain individual differences. Subjective well-being and personality have been studied, especially in the big five domains. All Big Five traits (extraversion, agreeableness, openness, neuroticism, and conscientiousness) have been shown to be related to life satisfaction [2,6,12], but the strongest correlates of satisfaction with life were neuroticism (negative) and conscientiousness (positive; [12,13]). In two recent meta-analyses, openness was unrelated to satisfaction with life, and the strongest effect sizes were in descending order: neuroticism (negative), extraversion, conscientiousness, and, finally, agreeableness [5,14]). Thus, extraversion was more strongly related with life satisfaction than conscientiousness in comparison to previous studies. These studies reveal the importance of considering personality when examining the relationship between recreation specialization and life satisfaction, which will be discussed in the following sections.

### 1.2 Recreation specialization

Recreation specialization is a concept in the field of leisure and recreation studies that refers to the extent to which an individual is engaged in leisure activities that match their interests and abilities [15]. Basically, recreation specialization is linked to a leisure career concept [15,16]. The beginner starts with a low level of knowledge, commitment, and behavior, and the leisure activity is not that central to one's lifestyle [17]. However, across time, people become more engaged, knowledgeable, and involved, so that they move from novices to experts [18]. This career can also be viewed from the serious leisure perspective where people can be interested sporadically into an activity but can also follow a given leisure interest rather in a professional manner [16]. The concept of recreation specialization has been studied in relation to a number of outcomes, including satisfaction with life, physical health, and well-being, as well as the development of personal skills and abilities. Recreation specialization is viewed as a multidimensional construct that can be influenced by a range of individual, social, and environmental factors [15,[19], [20], [21]]. Recreation specialization is measured in the components i) behavior, ii) skill/knowledge, and iii) commitment [15,20]. Knowledge refers to specific knowledge of a given activity, for instance, by being able to identify different kind of bird species without any help in the case of birdwatching. The behavior dimension is concerned with the number of field trips and outings, and the replacement value of one's equipment, or with the number of birds on a list (e.g., a life list, that counts all bird seen in one's lifetime). Commitment or centrality to lifestyle is a psychological component that measures the importance of the hobby for a person. Recently, support was found for the career development in German birders [18]. Skill/knowledge increased steadily across the career stages, while behavior increased and then remained on a stable level. Commitment, however, did not change during the career progression [18]. These dimensions of recreation specialization might be related to life satisfaction, which will be the focus of the following section.

### 1.3 Life satisfaction and recreation specialization

Leisure activity, including sports and pursuing a hobby, has been positively related to different concepts of well-being, such as quality of life, satisfaction with life, and subjective well-being [3,4,[22], [23], [24]]. Also, recreation specialization or serious leisure has been linked to satisfaction with life according to the theory of subjective well-being proposed by Diener et al. ([2]; see also [3,25]). In fact, satisfaction with life and leisure specialization may be related with each other. Therefore, serious leisure participation fulfils an individual's psychological need, which can positively affect subjective well-being [24]. Moreover, the leisure career aspect that describes skill progression and knowledge development during time [18,21], and occupational career in parallel have a similar positive effect on satisfaction with life [26]. In their meta-analysis, Kuykendall et al. [22] reported strong evidence for the positive association between leisure engagement and subjective well-being. Moreover, Spiers and Walker [8] suggested that the achievement component in leisure may be important for life satisfaction. This means that the measure of skill/knowledge in recreation specialization may be related to life satisfaction. However, the relationship between knowledge and satisfaction with life has been largely neglected (exception: [27]). In Matsumoto et al.'s [27] study, knowledge was the most important statistical direct predictor of happiness.

Tian et al. [3] showed that recreation specialization in runners was directly related to life satisfaction, with all dimensions: behavior (*β* = 0.15), cognition (*β* = 0.35), affect (*β* = 0.28), and psychological commitment (*β* = 0.59). Lee and Hwang [25] found that 3 out of 18 dimensions of serious leisure were significant correlates of subjective well-being (personal enrichment, self-expression of individual, self-gratification-enjoyment). And in accordance, there was a significant relationship between level of involvement in serious leisure and life satisfaction [28]. Therefore, a relationship between recreation specialization, especially the achievement dimension, and satisfaction with life is also expected in birders. The following section discusses the link between recreation specialization and the second variable predicting life satisfaction in our study, personality.

### 1.4 Personality and recreation specialization

Empirical studies have investigated the relationship between various personality traits, such as the Big Five (openness, conscientiousness, extraversion, agreeableness, and neuroticism) and specific recreational activities [29]. In general, differences in personality exist among different types of leisure activity (e.g., between model builders and artists [30]). For example, social leisure activities were linked to extraversion, and performing arts to openness for new experience [29]. Further studies showed that physically active leisure is related to satisfaction with life [31], which has usually been examined in sports. Here, it should be taken into account that birdwatching is also considered a physically active leisure activity with a cardio-vascular effect comparable to walking [32]. As birding takes place outdoors, the positive influence of nature [33] may also play a role. Sato et al. [31] studied runners and found that conscientiousness was positively associated with attraction, centrality to lifestyle, and self-expression. Openness to experience was also positively associated with self-expression, while extraversion, agreeableness, and emotional stability were not associated with running attraction, centrality, and self-expression [31]. Their study was based on a measure more related to the centrality of lifestyle construct, and it therefore differs in parts from the construct used in our present study, because we included the skill/knowledge dimension, as well as behavior. Similarly, Ong et al. [9] established a relationship between personality and experience in an activity (scuba diving) with attitude. In conclusion, recreation specialization and the Big Five domains of personality should be related although clear empirical evidence is scarce. Therefore, this study aims to contribute to this research question. A special focus might be placed on conscientiousness as this personality dimension is most strongly related to grit [34] and grit is characterized by long-term stamina and sustained effort and interest in the activity, often pursuing long-term goals [34]. In a similar manner, perseverance of effort was positively associated with attraction, centrality, self-expression, and life satisfaction [1].

### 1.5 Current study

In this study, we addressed the relationship between satisfaction with life, recreation specialization, and personality in a comprehensive manner. Previous studies ware mainly based on sports and physical activity [25], but no study was done in birders so far. Birdwatching, however, requires some activity in nature, but to a less strenuous extent as sports. Thus, birding differs in some respects from other outdoor recreational activities, because it is usually less “sportive”, but includes activities in nature and has also a cognitive component [35]. Although cognitive skills are needed (species knowledge, recognition), these skills are trainable. Sometimes birding is described as the most scientific of sports or the most sporting of science [36]. Therefore, recreation specialization is a different concept than personality because it undergoes some kind of career progress [18]. While personality remains difficult to alter, skill/knowledge, behavior, and commitment can significantly change, which has been shown in the context of acquiring skills in music [37].

As research questions, we wanted to explore whether there is a relationship between recreation specialization and satisfaction with life, and if this is the case, which dimension of recreation specialization is related to life satisfaction. Further, given the relationship between personality, recreation specialization, and life satisfaction, we wanted to study whether the relation between recreation specialization and life satisfaction is fully or partially mediated by personality using mediation analyses.

## 2 Materials and methods

### 2.1 Participants

In our study, 555 birders (*M*_age_ = 49.11 years, *SD*_age_ = 17.14 years, *Mdn*_age_ = 52.0 years, *R*_age_ = 12–88 years) from German-speaking countries (Germany = 97.3 %, Austria = 2.3 %, Switzerland = 0.4 %) filled out an online survey. One hundred and seventy of these participants stated that they were female, whereas 380 of them reported being male. Two participants chose the category diverse, while three of the participants did not want to make a statement about their gender. About 70 % of the participants had a university degree. The birdwatchers were recruited through the e-mail distribution lists and websites of German ornithological associations. In accordance with German data protection guidelines, participants were informed about the voluntary and anonymous nature of their participation as well as the handling and processing of their data. In addition, their informed consent was obtained. For their participation, the birdwatcher received a voucher of five Euro and had the chance to participate in a raffle of books about birdwatching and behavioral biology. The study was granted permission by the ethics committee of the 10.13039/501100005721University of Bielefeld (2021-121 from May 21, 2021).

### 2.2 Measures

The questions regarding recreation specialization were presented first, and life satisfaction was examined before the personality dimensions. The questionnaires used are presented in the following sections.

#### 2.2.1 Recreation specialization

Recreation specialization was measured with previously established scales [20]. Recreation specialization contains three dimensions: skill/knowledge is based on items that ask for the number of bird species one is able to identify by sight without help (up to 25, 26–45, 46–100, 101–250, 251–500, >500), the number of bird species one is able to identify by sound (up to 5, 6–10, 11–25, 26–80, 81–150, >150), and a self-classification item ranging from novice (1) to expert (5). Cronbach's α was 0.88. The behavior dimension was measured by the number of birding excursions per year (at least 2 km away from home: scaled from none, 1–2, 3–10, 11–35, >35); number of days with birding activity (none, <10, 11–30, 31–70,71–200, >200). Cronbach's α was 0.62. Psychological commitment was measured by three questions on a five-point Likert scale from "fully disagree" (1) to "fully agree" (5). These items state that other leisure activities are less interesting, that birding is preferred in comparison to every other leisure activity, and that if one couldn't go birding, he/she would not know what to do else. Cronbach's α was 0.78.

#### 2.2.2 Personality (big five Inventory-10)

The Big Five Inventory-10 scale proposed by Rammstedt and John [38] was used in the German version. The BFI-10 consists of 10 items, two for each dimension of personality (neuroticism, extraversion, openness, agreeableness, conscientiousness). Each of the dimensions is represented by a positive and a negative item recorded. Participants responses were collected by a five-point rating scale from "does not apply at all" (1) to "applies completely" (5). The brief measure was used because of time constraints and compliance of the online survey.

#### 2.2.3 Satisfaction with life

To measure satisfaction with life, we used one global item proposed by Beierlein et al. [39]: "How satisfied are you currently, all in all, with your life?". We used the item coding from "not at all satisfied" (1) to "completely satisfied" (10). The item was used because of time constraints of the online survey.

#### 2.2.4 Statistical analysis

We used correlational analysis (Pearson's correlation) to study the bivariate relationships between life satisfaction and personality/recreation specialization, and between personality and recreation specialization. To address the complex relationship further, we studied it by mediation. Mediation analyses was used to assess whether the relationship between recreation specialization onto life satisfaction was a direct path or whether it was mediated by personality. In this case, mediation analysis tries to explain the relationships between variables. Mediation analyses were performed using the PROCESS macro by Hayes [40], which uses ordinary least squares regression to calculate unstandardized path coefficients for total, direct, and indirect effects. The results were obtained based on 5000 bias-corrected bootstrapped samples to compute the confidence intervals and inferential statistics. Effects were regarded as significant when the confidence interval did not include zero. All statistics were carried out with SPSS 28.

## 3 Results

Descriptive statistics are given in Table 1.

**Table 1:** Descriptive statistics of the sample. N = 555 for all variables except Satisfaction with Life (N = 554).

	*M*	*SD*
Extraversion	2.97	1.01
Agreeableness	3.35	0.80
Conscientiousness	3.83	0.81
Neuroticism	2.68	0.93
Openness	3.58	1.02
Skill/Knowledge	3.73	1.06
Behavior	4.03	1.00
Commitment	2.77	0.92
Satisfaction with Life	7.96	1.54

Satisfaction with life was positively correlated with extraversion, agreeableness, and conscientiousness, as well as negatively with neuroticism (Table 2). Openness was unrelated to satisfaction with life. Concerning recreation specialization, skill/knowledge and behavior were positively related to satisfaction with life, but not psychological commitment. This means that birders with a higher knowledge and with more field day outings scored higher on satisfaction with life (Table 2).

**Table 2:** Correlations between satisfaction with life, personality, and recreation specialization. E = Extraversion, A = Agreeableness, C = Conscientiousness, N = Neuroticism, O = Openness, SWL = Satisfaction with Life.

		E	A	C	N	O	Skill/Knowledge	Behavior	Commitment
E	Pearson's *r*	1							
	*p*								
A	Pearson's *r*	.067	1						
	*p*	.114							
C	Pearson's *r*	.185	.105	1					
	*p*	<.001	.013						
N	Pearson's *r*	−.200	−.144	−.220	1				
	*p*	<.001	<.001	<.001					
O	Pearson's *r*	.239	.062	.103	−.042	1			
	*p*	<.001	.148	.015	.329				
Skill/Knowledge	Pearson's *r*	.066	.082	.121	−.138	−.132	1		
	*p*	.123	.052	.004	.001	.002			
Behavior	Pearson's *r*	−0.003	.029	.069	−.062	−.094	.588	1	
	*p*	.944	.497	.102	.147	.027	<.001		
Commitment	Pearson's *r*	−.067	.038	−.034	.079	−.165	.306	.388	1
	*p*	.117	.377	.423	.063	<.001	<.001	<.001	
SWL	Pearson's *r*	.214	.165	.257	−.351	.045	.157	.123	−.036
	*p*	<.001	<.001	<.001	<.001	.288	<.001	.004	.394

Recreation specialization was also correlated with personality (Table 2), especially the dimension of skill/knowledge to conscientiousness, neuroticism, and openness. Higher conscientiousness was related to higher recreation specialization (skill/knowledge). Openness was negatively related to three dimensions of recreation specialization; thus, higher specialized birders seem more focused on fewer topics. Neuroticism was negatively related to skill/knowledge, thus, higher specialized birders in terms of skill/knowledge scored lower on neuroticism.

### 3.1 Mediation analysis

**Fig. 1:**
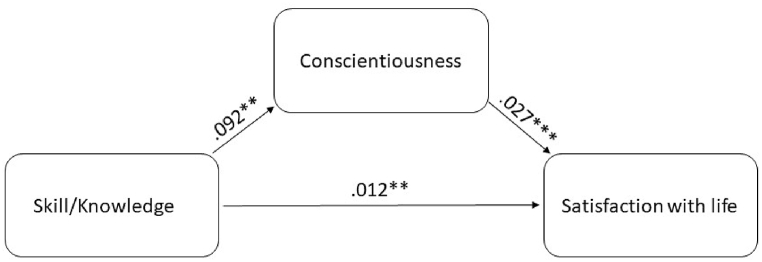
Mediation analysis between the skill/knowledge dimension for recreation specialization and satisfaction with life via the pathway conscientiousness.

For the mediation analysis, only variables that were significantly intercorrelated were used. Therefore, we assessed the mediational effect of conscientiousness in the relationship between skill/knowledge and satisfaction with life in order to test whether the link between skill/knowledge and satisfaction with life remains or disappears when conscientiousness was included as personality correlate of satisfaction with life. In the first step, a total effect of skill/knowledge on satisfaction with life was observed, *B* = .014, *p* < .001, 95%-CI [0.007,0.022]. After entering the mediator into the model, the path from skill/knowledge was significantly related to the mediator (conscientiousness), *B* = 0.092, *p* < .001, 95%-CI [0.029,0.157], which, in turn, was significantly related to satisfaction with life, *B* = 0.027, *p* = .001, 95%-CI [0.017,0.037]. Finally, we found that the relationship between skill/knowledge and satisfaction with life was partially mediated by conscientiousness *B* = .012, *p* = .001, 95%-CI [0.004,0.020; see Fig. 1], but skill/knowledge has a direct path on satisfaction with life.

**Fig. 2:**
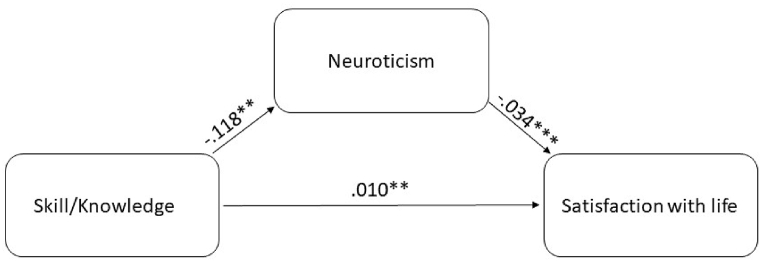
Mediation analysis between the skill/knowledge dimension for recreation specialization and satisfaction with life via the pathway neuroticism.

Further, we investigated whether neuroticism mediates the relationship between skill/knowledge and satisfaction with life. The initial analysis showed that there was a significant path from skill/knowledge on satisfaction with life (*B* = .014, *p* < .001, 95%-CI [0.904, 0.983]). When including neuroticism as a mediator in the model, we found that skill/knowledge was significantly related to neuroticism (*B* = −0.119, *p* < .001, 95%-CI [-0.192, −0.045]), which, in turn, was related to satisfaction with life (*B* = −0.034, *p* < .001, 95%-CI [-0.042, −0.025]). Finally, we found that the relationship between skill/knowledge and satisfaction with life was partially mediated by neuroticism (*B* = .010, *p* < .001, 95%-CI [0.002,0.018], Fig. 2). Based on our results, we can conclude that skill/knowledge is directly and positively related to satisfaction with life. Although neuroticism weakens this effect, the mediating role of neuroticism is small, indicating that the link between skill/knowledge and satisfaction with life is not solely due to the presence of neuroticism.

**Fig. 3:**
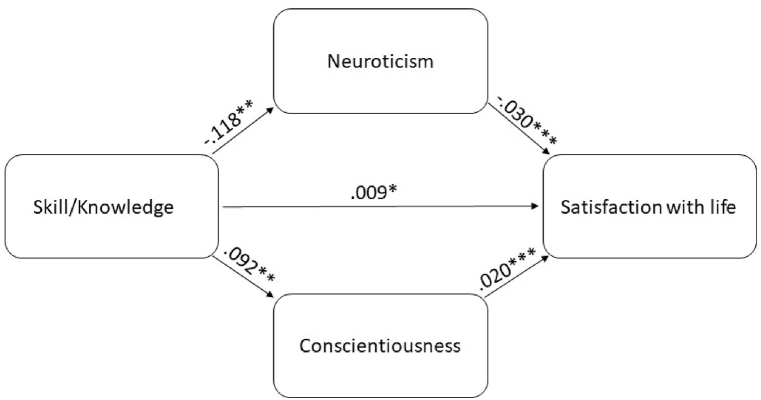
Mediation analysis between the skill/knowledge dimension for recreation specialization and satisfaction with life via the two pathways neuroticism and conscientiousness.

Finally, we entered both mediators into the model. Mediation analysis indicated that the relationship between skill/knowledge and satisfaction with life was mediated by neuroticism and conscientiousness but remained significant at a lower significance level (p < .05). The total effect of skill/knowledge on life satisfaction was 0.014 (p < .001, 95%-CI [0.006, 0.002]), and the direct effect (the path through mediators) became significantly smaller but remained significant (B = −0.009, p < .05, 95%-CI [0.001, 0.006]; Fig. 3).

## 4 Discussion

In the current study, we combined recreation specialization and personality as correlates of satisfaction with life. With this approach, we add to the knowledge that recreation specialization is linked with life satisfaction, for the first time in a non-sporting nature-based outdoor activity [4,28,31]. The behavior and the cognitive (skill/knowledge) dimensions were both related to satisfaction in life. Although the behavior dimension in birding is conceptually similar to the behavior dimension in marathon runners, there are still differences in the strenuousness of the activities, and birding walks are far less demanding in most cases compared to marathon training. This is an important difference because older adults with more physical limitations can still go out for a bird walk, and birding is one of the few outdoor activities that can be excelled by children, adults, and grandparents simultaneously [41]. The results are further in line with previous work where an exposition to nature has been shown to improve well-being and health, and more field trips and days out were related with a higher satisfaction [33]. Thus, if people become unable to run marathon or master other physical activities, birding can still be an alternative.

In their model, Lee and Scott [42] suggested that as individuals became increasingly specialized, they obtain benefits that outweigh any costs during their leisure specialization or leisure career [18]. Especially, skill/knowledge increases throughout the leisure career [18], therefore, benefits from this activity still increase with leisure career duration, and, in turn, this knowledge is related to satisfaction with life. Overcoming obstacles is as important in birding as in sports [43], showing another similarity between both leisure activities. The further developed a birding career is, the more birders spent time outdoors even during adverse weather conditions such as wind, rain, and cold [43]. By managing and mastering these adverse conditions, this behavior may positively influence life satisfaction via pathways, such as experiencing competence [24]. Matsumoto et al. [27] showed an important relationship between knowledge and life satisfaction. Knowledge can be associated with mastery, which is itself an important aspect of leisure satisfaction [24], and may, in turn, is related to life satisfaction. In the elite birding segment, birders are able to identify nearly 500 different species by visual cues, and nearly 200 by acoustic cues [35]. Thus, such a high specialization can possibly contribute to life satisfaction. However, it will become an important topic to separate the different aspects of well-being from each other, for instance, satisfaction with life from psychological well-being.

We conclude that skill/knowledge has a direct and positive relationship with satisfaction with life. When we entered conscientiousness and neuroticism as mediators in the model the coefficient weakened, but the mediation was small, so we infer that the presence of the direct association between skill/knowledge and satisfaction with life is not solely due to the mediating roles of conscientiousness and neuroticism.

Openness to new experiences was negatively related to skill/knowledge, behavior, and commitment: higher specialized birders seem more narrow-minded or more focused in their interests. Thus, the development of a leisure career [18,42] may lead to a higher mastery level, but probably on the costs of losing interest in other activities. Such a relationship is not easy to judge, but focusing on a complex leisure activity may reduce the time and capacity available for other interests. On the one hand, birdwatching can be an extraordinarily cognitively demanding task by identifying hundreds of different bird species without any aid (by sight and sound), so it needs experience and training. On the other hand, birdwatching may also attract more focused people who prefer to specialize in one activity rather than having a broader interest. As the direction and causality is not yet solved, this might be an interesting venue for further research. Skill/knowledge was also positively related to conscientiousness, which may be related to the fact that observations of special and rare species need a thorough assessment and documentation. However, as birdwatching is a cognitive demand (or achievement), it may be just the relationship between conscientiousness and academic achievement (e.g., [44]) that is mirrored in the relationship of conscientiousness and higher birding specialization. Also, neuroticism as a personality dimension distracts from specializing in an activity in a general manner because it is a tendency to respond with negative emotions to threat, frustration, or loss [45]. Therefore, we assume that neuroticism is the cause for lower engagement in a demanding activity.

Many previous studies have used different variables to measure leisure engagement, such as mainly measuring behavior [22], as well as more psychological constructs like psychological commitment [46], based on serious leisure theory. However, the recreation specialization concept [15] has the benefit of measuring behavior, knowledge, and involvement/centrality to lifestyle within one questionnaire [19,20]. This gives an advantage when studying satisfaction with life, recreation specialization and personality in one study.

The correlations between satisfaction with life and Big Five are very similar to meta-analytical results [12,13]. Openness was unrelated to life satisfaction, and the strongest predictor was neuroticism (negative) followed by conscientiousness, extraversion and, finally, agreeableness. In contrast, Steel et al. [14] and Anglim et al. [5] found extraversion as more important than conscientiousness. Nevertheless, the results are largely in accordance with previous work. As we studied a special population and not a representative sample, we strongly suggest comparing personality dimensions between birders and the general population to assess possible differences that may be ascribed to this leisure activity.

### 4.1 Limitations

As a limitation, we used brief measures to achieve a higher compliance in our online survey. Therefore, future studies may apply longer Big Five versions and a full satisfaction with life scale, like the Satisfaction with Life Scale [47]. However, data from our respondents are difficult to study because they are widely scattered across the geographical region. An online procedure is one of the best attempts to cover birders in as many regions as possible and to secure a high compliance, short measures are advantageous.

### 4.2 Outlook

Further studies should apply a variety of measures concerning leisure activities, including psychological constructs [17], and measures of specialization [15]. Concerning more objective tasks, tests could be carried out, for instance, with identification ability in a standardized test situation, because previous research showed that mental abilities can be related to objectively measured psychomotor tasks [48]. In addition, personality dimensions could be enriched by focusing on grit [34], especially in birding, because this sometimes requires sustained effort and interest in the activity [34]. Also, other personality inventories might be used to study the relationship further.

## 5 Conclusion

As a conclusion, physical active outdoor recreational activity, such as birding, has a positive impact on life satisfaction, and this is mainly based on the cognitive component, with a high knowledge related to a higher life satisfaction.

## Data availability statement

The datasets is publicly available on the Open Science Framework under https://osf.io/3jpnx/.

## Funding

We acknowledge support by Open Access Publishing Fund of University of Tübingen.

This project is part of the “Qualitätsoffensive Lehrerbildung”, a joint initiative of the Federal Government and the *Länder* which aims to improve the quality of teacher training. The program is funded by the 10.13039/501100002347Federal Ministry of Education and Research (funding code: 01JA1908). The authors are responsible for the content of this publication.

## Ethics statement

The study was granted permission by the ethics committee of the 10.13039/501100005721University of Bielefeld (2021-121 from May 21, 2021). Respondents were informed about the study, data protection rules following European law and had to actively click on a “Yes” to start the participation.

## CRediT authorship contribution statement

**Christoph Randler:** Conceptualization, Data curation, Formal analysis, Investigation, Project administration, Writing – original draft. **Arash Rahafar:** Formal analysis, Methodology, Writing – original draft. **Nadine Großmann:** Conceptualization, Data curation, Investigation, Methodology, Writing – review & editing.

## Declaration of competing interest

The authors declare that they have no known competing financial interests or personal relationships that could have appeared to influence the work reported in this paper.
